# Advanced MR Technique for the Prediction of a Complete Response Following Neoadjuvant Chemoradiotherapy in Patients with Locally Advanced Rectal Cancer: A Comparative Study Between DWI *b*2000 and *b*1000

**DOI:** 10.3390/jpm16100537

**Published:** 2026-10-21

**Authors:** Geon Lee, Seung Ho Kim, Mingyu Kim, Dajung Kim, Hye Ri Kim

**Affiliations:** Department of Radiology, Haeundae Paik Hospital, Inje University College of Medicine, Busan 48108, Republic of Korea

**Keywords:** rectal cancer, magnetic resonance imaging (MRI), diffusion-weighted imaging (DWI), neoadjuvant chemoradiotherapy (nCRT), complete response (CR)

## Abstract

**Background/Objectives**: From the viewpoint of personalized medicine, it is important to properly select patients with a complete response (CR) after neoadjuvant chemoradiotherapy (nCRT) because they are candidates for “watch-and-wait” management in locally advanced rectal cancer (LARC). The aims of this study were to evaluate the added value of diffusion-weighted imaging (DWI) *b*2000 to high-resolution T2-weighted images (HR-T2WI) and to compare the diagnostic performance of DWI *b*2000 and *b*1000 for assessing a CR in patients with LARC after nCRT. **Methods**: This retrospective study included 55 patients (male: 40, female: 15; mean age: 60.1) who underwent pre- and post-nCRT 3T MRI, including DWI_b1000_ and DWI_b2000_. Two blinded radiologists independently assessed the likelihood of a CR using a five-point scale across three image sets: T2-weighted images (T2WI), T2WI + DWI_b1000_, and T2WI + DWI_b2000_. T2WI were provided because DWI is not used as a standalone sequence in clinical practice when evaluating the therapeutic response to nCRT. A crossover review of DWI_b1000_ and DWI_b2000_ with a four-week interval was used to minimize recall bias. The reference standard of a CR was histopathologic examination or clinical remission in cases of watch-and-wait management. For each reader, diagnostic performance was compared across three image sets using pairwise comparisons of the receiver operating characteristic (ROC) curves. **Results**: The study population comprised 19 patients with a CR and 36 patients with residual tumor. For both readers, adding DWI_b2000_ significantly enhanced diagnostic performance compared to T2WI alone (AUC for reader 1: 0.918, 0.806, *p* = 0.0288; AUC for reader 2: 0.868, 0.768, *p* = 0.0414, respectively), likewise, DWI_b1000_ (for reader 1: 0.955, *p* = 0.0015; for reader 2: 0.863, *p* = 0.0162). However, no significant difference in diagnostic performance was found between DWI_b1000_ and DWI_b2000_ for each reader (for reader 1: *p* = 0.0931; for reader 2: *p* = 0.8936). **Conclusions**: Adding DWI_b2000_ to T2WI improved diagnostic performance for identifying a CR after nCRT compared to T2WI alone. No statistical difference was detected between DWI_b1000_ and DWI_b2000_.

## 1. Introduction

Magnetic resonance imaging (MRI) remains the cornerstone imaging modality for staging and restaging of rectal cancer [1]. The traditional standard of care for locally advanced rectal cancer (LARC) consists of neoadjuvant chemoradiotherapy (nCRT) followed by total mesorectal excision [2]. However, given the significant morbidity and quality-of-life implications associated with surgical treatment, there has been increasing adoption of non-operative management (the “watch-and-wait” strategy) for patients who achieve a clinical complete response (cCR) [3,4,5,6]. In the current era of total neoadjuvant therapy (TNT), which yields higher rates of cCRs of up to 30%, the accuracy of restaging MRI has become even more critical for selecting appropriate candidates for an organ-preserving strategy [7,8,9,10].

From the viewpoint of personalized medicine, proper selection of patients who have achieved a cCR is a key point for successful non-operative management. The primary advantage of this approach lies in its ability to maintain oncologic safety, with survival rates comparable to those of surgical resection, while avoiding the substantial morbidity associated with surgery [10,11,12]. However, the successful implementation of this strategy requires frequent observation using a multimodal surveillance protocol combining high-resolution rectal MRI with serial endoscopic assessment to ensure the early detection of local growth or microscopic residual disease [11].

High-resolution T2-weighted imaging (HR-T2WI) serves as the foundation for evaluating the treatment response of rectal cancer by providing detailed anatomical and morphological information of the tumor bed [13,14]. However, differentiating residual viable tumor from radiation-induced fibrosis and desmoplastic reactions with HR-T2WI remains a diagnostic challenge, as both can appear hypointense [15]. Diffusion-weighted imaging (DWI) addresses this challenging issue by providing functional information regarding tissue cellularity, thereby increasing sensitivity for residual tumor when added to HR-T2WI [14,16,17]. However, standard *b*-value (e.g., *b* = 1000 s/mm^2^) DWI still carries intrinsic limitations, as insufficient differentiation of residual tumor from fibrosis or desmoplastic reactions may lead to a false-negative interpretation of a CR, which may inappropriately disqualify complete responders after nCRT [15,18].

Ultra-high *b*-value DWI (*b* ≥ 1400 s/mm^2^) potentially overcomes this limitation by more effectively suppressing background signal intensity (SI) from benign tissues and fibrosis [19,20]. This imaging technique has been well established in prostate cancer, and DWI *b* ≥ 1400 s/mm^2^ is mandatorily recommended to satisfy technical requirements for optimal acquisition of MRI [21,22]. It is well known that ultra-high *b*-value DWI can increase specificity in detecting prostate cancer by suppressing SI from neighboring inflammatory or normal tissue [23]. In rectal cancer, previous studies have demonstrated that DWI_b2000_ improves tumor conspicuity and inter-observer agreement compared to DWI_b1000_ [24,25,26]. Despite these potential advantages, to the best of our knowledge, the added value of DWI_b2000_ to HR-T2WI has not been proven yet. Therefore, the aims of this study were to evaluate the added value of DWI_b2000_ to HR-T2WI and compare the diagnostic performance of DWI_b2000_ and DWI_b1000_ for assessing CRs in patients with LARC following nCRT.

## 2. Materials and Methods

This retrospective study was approved by our institutional review board, and the requirement for informed consent from individual patients was waived.

### 2.1. Patients and Selection Criteria

**Figure 1:**
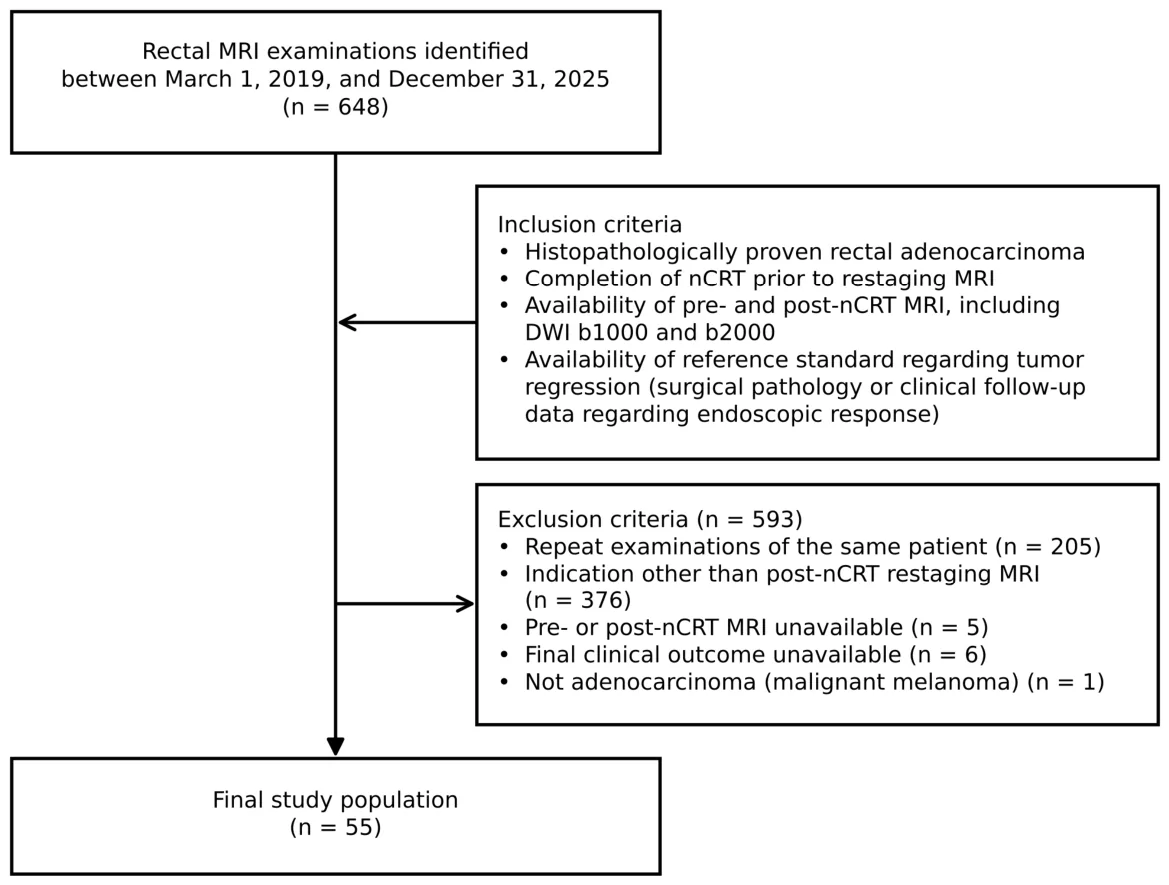
Flowchart of the patient enrollment process.

We retrospectively searched our institution’s Picture Archiving and Communication System (PACS, M6, Infinitt Healthcare, Seoul, Republic of Korea) for patients who underwent rectal MRI between March 2019 and December 2025, and identified a total of 648 rectal MRI examinations. Patients were eligible for inclusion if they met the following criteria: (1) histopathologically confirmed rectal adenocarcinoma; (2) completion of nCRT prior to restaging MRI; (3) availability of pre- and post-nCRT MRI including DWI_b1000_ and DWI_b2000_; and (4) availability of a definitive reference standard regarding tumor regression, defined by either a surgical pathology specimen or clinical follow-up data regarding endoscopic response. All enrolled patients received nCRT with a fluoropyrimidine-based regimen, including capecitabine, FOLFOX, and FOLFIRI. Exclusions were made for exams for indications other than rectal cancer restaging, duplicate studies on the same patient, lack of an appropriate reference standard, or pathology other than rectal adenocarcinoma. Finally, 55 consecutive patients were included for image interpretation and subsequent statistical analysis. The patient enrollment process is summarized in Figure 1.

### 2.2. MRI Protocol

All MR images were acquired with a 3-Tesla MR scanner (Achieva; Philips Medical Systems, Best, The Netherlands) using a torso coil (SENSE; USA Instruments, Gainesville, FL, USA). The protocol included HR-T2WI in three planes and multi-*b*-value DWI (*b* = 0, 100, 1000, and 2000 s/mm^2^). For sufficient distension of the rectal canal, endorectal filling with 80 to 100 mL of ultrasound gel (Ecosonic, Sanipia, Jeonbuk, Republic of Korea) was performed before the examination. Detailed scan parameters are summarized in Table 1.

### 2.3. Image Interpretation

Two radiologists with 15 and 3 years, respectively, of clinical experience in interpreting rectal MRI independently evaluated the images while blinded to the clinical, endoscopic, and pathologic results. Initially, each reader evaluated the likelihood of a CR by reviewing pre- and post-nCRT T2WI images only in order to investigate the baseline performance of the commonly used T2WI. Readers then reviewed the combined sets of T2WI and DWI (either DWI_b1000_ or DWI_b2000_) in two separate sessions. Using a crossover design, the cohort was randomized such that each reader initially assessed half of the cases with T2WI + DWI_b1000_ and the other half with T2WI + DWI_b2000_. In the final session, each reader assessed the remaining DWI *b*-value images that were not previously reviewed alongside T2WI. To minimize recall bias, sessions were scheduled at four-week intervals. For each session, pre-nCRT T2WI and DWI were made available to the readers for baseline comparison and lesion localization. In the DWI sessions, readers were provided with the ADC map corresponding to the *b*-value being reviewed. Readers were blinded to the *b*-value labels during the DWI sessions.

For each examination, each reader assessed the likelihood of a CR using a 5-point Likert scale: 1, definitely not a CR; 2, probably not a CR; 3, possibly a CR; 4, probably a CR; 5, definitely a CR. The scoring criteria were determined by integrating morphological findings on T2WI with signal characteristics on DWI. On T2WI, the complete replacement of the intermediate SI of the tumor by dark SI was considered to represent a CR, whereas the presence of residual intermediate SI was considered to represent residual tumor [27]. On DWI, total disappearance of high SI at the original tumor site was regarded as a CR, whereas persistent focal high SI was defined as residual tumor [27]. When equivocal diffusion restriction made it difficult to distinguish residual tumor from post-treatment fibrosis or inflammation, readers adjusted their confidence scores accordingly.

### 2.4. Reference Standard

A complete response was defined as (i) a pathologic complete response (pCR) in patients who underwent surgery, or (ii) a sustained clinical complete response (cCR) in patients managed non-operatively. The reference standard for a pCR was established via histopathologic evaluation of the surgical specimen for patients who underwent resection. For surgical cases, pathological evaluation was conducted by a dedicated pathologist in accordance with the tumor regression grading (TRG) system of the American Joint Committee on Cancer (AJCC), 8th edition [28]. For patients managed non-operatively under the “watch-and-wait” strategy, a sustained cCR was defined as no endoscopic, clinical, or radiologic evidence of regrowth for at least 12 months after restaging MRI. Conversely, residual tumor was defined by positive endoscopic findings or a positive biopsy result. The decision to enter non-operative management was made by a multidisciplinary team after discussion of the digital rectal examination, endoscopy, and restaging MRI, including HR-T2WI, DWI_b1000_, and DWI_b2000_.

### 2.5. Statistical Analysis

Diagnostic performance for the prediction of a CR was evaluated using receiver operating characteristic (ROC) curve analysis. For each reader, the area under the curve (AUC) was calculated across the three image sets (T2WI, T2WI + DWI_b1000_, and T2WI + DWI_b2000_). Pairwise comparisons of the ROC curves were performed using DeLong’s test to assess statistical significance. Diagnostic accuracy, sensitivity, specificity, and positive and negative predictive values were calculated by dichotomizing the confidence scores, with scores of 3 or higher pre-defined as positive for the diagnosis of a CR. In addition, a sensitivity analysis restricted to the surgical cohort was performed. Statistical significance was assessed across the image sets for each reader using McNemar’s test.

Quadratic-weighted kappa values were calculated to assess the inter-observer agreement in terms of confidence levels for the prediction of a CR across the three image sets between the two readers. Kappa (*k*) values were interpreted as follows: 0.01–0.20 as slight agreement; 0.21–0.40 as fair agreement; 0.41–0.60 as moderate agreement; 0.61–0.80 as substantial agreement; and 0.81–1.00 as almost perfect agreement. All statistical analyses were performed using MedCalc software (version 20.113; MedCalc Software, Mariakerke, Belgium). A *p* value of less than 0.05 was considered to indicate a statistically significant difference.

## 3. Results

### 3.1. Patient Demographics

A total of 55 consecutive patients (40 males, 15 females; mean age, 60.1 years) were enrolled in this study. Based on the reference standard, 19 patients (35%) achieved a CR, consisting of two patients with a pCR (TRG 0) and 17 managed under a watch-and-wait strategy with a sustained cCR. For the patients managed non-operatively, the median follow-up period was 20 months (IQR: 14.4 to 28.0), and six patients were followed for more than 24 months. The remaining 36 patients (65%) had residual tumor, including nine with a near-CR (TRG 1), 19 with a partial response (TRG 2), and eight with a poor response (TRG 3). For the 38 patients who underwent surgical resection, the final pathologic stages were ypT0 (*n* = 2), ypT1 (*n* = 1), ypT2 (*n* = 13), ypT3 (*n* = 20), and ypT4 (*n* = 2). Detailed demographic and clinico-pathologic characteristics are summarized in Table 2.

### 3.2. Comparison of Diagnostic Performance

**Figure 2:**
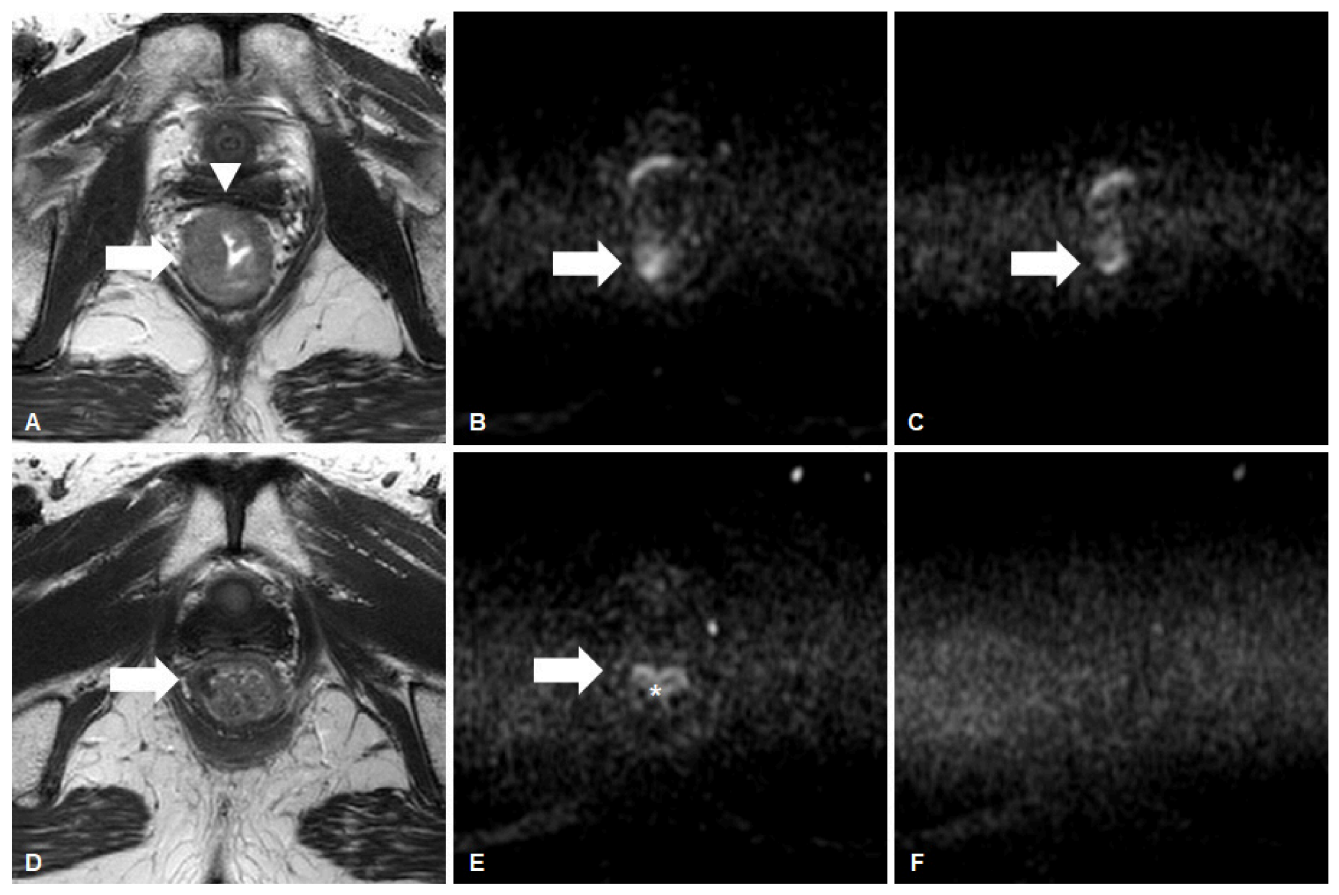
MR images from a 56-year-old female with lower rectal cancer showing added value of DWI_b2000_ over DWI_b1000_ for predicting complete response (CR): (**A**) Pre-nCRT T2WI shows eccentric wall thickening in the lower rectum (arrow) invading the anterior mesorectal fascia (arrowhead). (**B**,**C**) Pre-nCRT DWI_b1000_ and DWI_b2000_, respectively, show diffusion restriction in the tumor (arrows). (**D**) Post-nCRT T2WI shows dark signal intensity (SI) at the tumor bed, suggesting fibrotic conversion of the tumor (arrow). (**E**) Post-nCRT DWI_b1000_ shows focal residual high SI at the tumor bed (arrow) with intraluminal high SI (asterisk). (**F**) The focal high SI at the tumor bed and intraluminal high SI are no longer observed on post-nCRT DWI_b2000_. Diagnosis of CR was made, which was based on multidisciplinary team approach consisting of digital rectal examination, endoscopy, and restaging MRI. This patient was managed non-operatively and maintained clinical CR. Confidence scores (T2WI/T2WI + DWI_b1000_/T2WI + DWI_b2000_): reader 1—3/4/5; reader 2—2/2/4.

**Figure 3:**
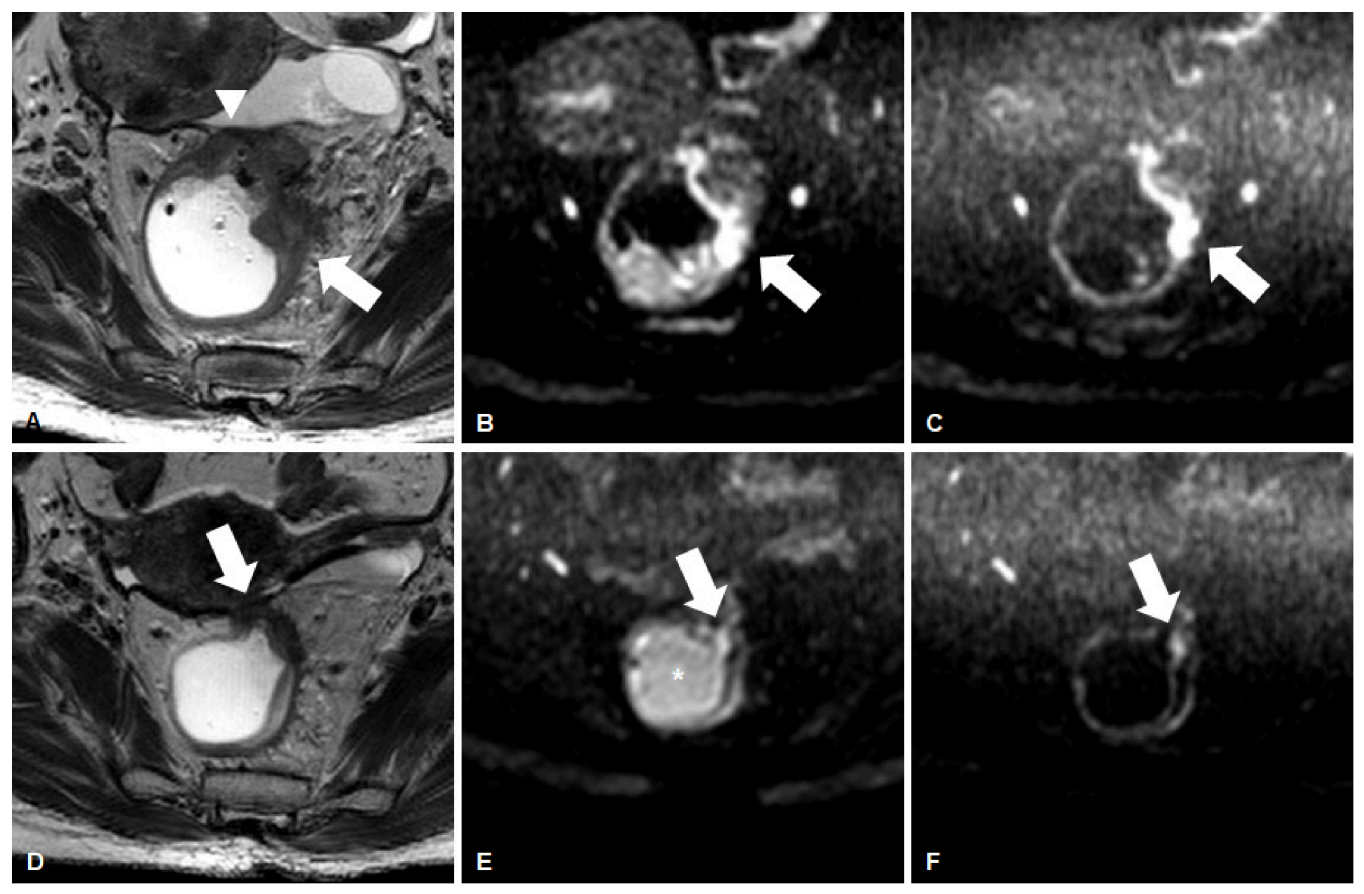
MR images from a 48-year-old female with mid rectal cancer and pathologic stage of ypT3N0, demonstrating added value of DWI_b2000_ over DWI_b1000_ for identifying residual tumor: (**A**) Pre-nCRT T2WI shows an ulcerofungating mass in the mid rectum (arrow) with direct extension to the anterior peritoneal reflection (arrowhead). (**B**,**C**) Pre-nCRT DWI_b1000_ and DWI_b2000_, respectively, show strong diffusion restriction in the tumor (arrows). (**D**) Post-nCRT T2WI demonstrates decreased tumor size with conversion to dark signal intensity (SI), suggestive of fibrotic replacement of the tumor (arrow). (**E**) Post-nCRT DWI_b1000_ shows equivocal high SI in the tumor surface (arrow), although surrounding high SI from intraluminal gel (asterisk) reduces the conspicuity of residual tumor. (**F**) Post-nCRT DWI_b2000_ shows a well-defined residual focal high SI in the tumor bed (arrow), resulting from sufficient suppression of intraluminal high SI. The patient was surgically treated following nCRT and had a pathologic tumor regression grade of 2 (partial response), indicating evident tumor regression but residual viable tumor beyond scattered single or small groups of cancer cells. Confidence scores (T2WI/T2WI + DWI_b1000_/T2WI + DWI_b2000_): reader 1—3/2/1; reader 2—2/2/1.

For both readers, the addition of DWI_b2000_ significantly improved diagnostic performance compared to T2WI alone (*p* < 0.05). For reader 1, the AUC for T2WI was 0.806 [95% confidence interval (CI): 0.677–0.900], which significantly increased to 0.918 (95% CI: 0.812–0.975) with T2WI + DWI_b2000_ (*p* = 0.0288). For reader 2, the AUC for T2WI was 0.768 (95% CI: 0.654–0.881), which also significantly increased to 0.868 (95% CI: 0.770–0.966) with T2WI + DWI_b2000_ (*p* = 0.0414). Likewise, for both readers, the addition of DWI_b1000_ significantly increased diagnostic performance (for reader 1: 0.955, *p* = 0.0015; for reader 2: 0.863, *p* = 0.0162). However, there was no significant difference between T2WI + DWI_b1000_ and T2WI + DWI_b2000_ (for reader 1: *p* = 0.0931; for reader 2: *p* = 0.8936). Although it was not statistically significant, there were several cases showing an advantage of DWI_b2000_ over DWI_b1000_. Representative examples where the addition of DWI_b2000_ to DWI_b1000_ provided additional benefit for predicting a CR or detecting residual tumor are provided in Figure 2 and Figure 3.

For reader 1, diagnostic accuracy increased significantly from 66% on T2WI to 91% on T2WI + DWI_b2000_ and 93% on T2WI + DWI_b1000_ (*p* = 0.0013 and *p* = 0.0007, respectively). Reader 2 showed a significant improvement in accuracy from 71% to 84% only with the addition of DWI_b2000_ (*p* = 0.0391). The diagnostic performance measures for each reader across the three image sets are summarized in Table 3.

In the subgroup of 38 patients who underwent surgery (two with a pCR and 36 with residual tumors), specificity was identical to that in the entire cohort (Table 4). Sensitivity is reported as counts only, as there were only two patients with a pCR in this group and sensitivity could not be reliably estimated.

### 3.3. Inter-Observer Agreement

Inter-observer agreement was substantial across all three image sets: for T2WI, *k* = 0.624 (95% CI: 0.468–0.779); for T2WI + DWI_b1000_, *k* = 0.639 (95% CI: 0.489–0.789); for T2WI + DWI_b2000_, *k* = 0.776 (95% CI: 0.636–0.915).

## 4. Discussion

Our results demonstrate that incorporating DWI_b2000_ into HR-T2WI significantly improved diagnostic performance for predicting a CR in patients with LARC following nCRT. This observation is consistent with the established role of DWI for providing functional information regarding tumor cellularity that complements morphological assessment provided by HR-T2WI. Previous studies have consistently reported that DWI_b1000_ improves the prediction of a CR and detection of residual tumor in rectal cancer restaging [16,29,30,31]. This complementary role is reflected in current recommendations, which advocate routine incorporation of DWI into rectal MRI protocols for therapeutic response assessment after nCRT [32].

Regarding the role of ultra-high *b*-value DWI, its clinical utility has already been established in prostate cancer, where acquisition of DWI with *b* ≥ 1400 s/mm^2^ is mandatorily recommended in current imaging protocols [21,22]. The use of ultra-high *b*-value DWI has been associated with improved specificity for tumor detection by reducing signals from inflammation and fibrosis, thus reducing false-positive interpretations. While the clinical utility of ultra-high *b*-value DWI has also been explored in rectal cancer, previous studies have largely focused on image quality assessment and tumor conspicuity [24,25,33]. Although previous studies have evaluated the diagnostic performance of ultra-high *b*-value DWI (*b*1500 and *b*2000) for detecting residual tumor in LARC after nCRT, they were limited by a relatively small study population [26] or a higher proportion of complete responders (54%) in the study cohort [24], which may have limited the generalizability of their observations. Unlike previous investigations, our study assessed the reader’s confidence level using a five-point Likert scale, providing a more convincing methodology beyond a binary classification of residual tumor as either present or absent [24,26].

When comparing the diagnostic performance of DWI_b2000_ with that of DWI_b1000_, we found no statistically significant difference in the overall diagnostic performance for predicting a CR. Our observation corresponds well with several previous investigations. Delli Pizzi et al. compared DWI_b2000_ and DWI_b1000_ for the detection of residual viable tumor in 41 restaging MRI scans following nCRT and reported no significant difference in diagnostic accuracy between the two *b*-values [24]. Similarly, a multi-reader study by Bates et al. compared DWI_b1500_ and DWI_b800_ for rectal cancer restaging after nCRT, and found no significant difference in the pooled sensitivity and specificity for the detection of residual tumor [26]. Collectively, these studies suggest that increasing the *b*-value beyond 1000 s/mm^2^ does not necessarily improve the diagnostic accuracy in the setting of rectal cancer restaging.

Although there was no statistically significant difference in terms of overall diagnostic accuracy between DWI_b1000_ and DWI_b2000_, we observed several instances in which DWI_b2000_ increased diagnostic confidence compared with DWI_b1000_. This observed benefit may be attributable to the increased tumor conspicuity associated with DWI_b2000_. Previous studies have reported that DWI_b2000_ provides improved tumor conspicuity with more effective suppression of signals from post-treatment inflammation and fibrosis [24,25]. The improved tumor conspicuity associated with DWI_b2000_ may explain the increased reader confidence in selected cases where diagnostic ambiguity caused by insufficient suppression of post-treatment desmoplastic reactions in DWI_b1000_ was effectively resolved with DWI_b2000_. In light of these findings, DWI_b2000_ may serve as a complementary sequence in a multi-parametric MRI protocol, in which improved tumor conspicuity can facilitate interpretation of true residual tumor within post-nCRT desmoplastic reactions, including inflammation and fibrosis.

**Figure 4:**
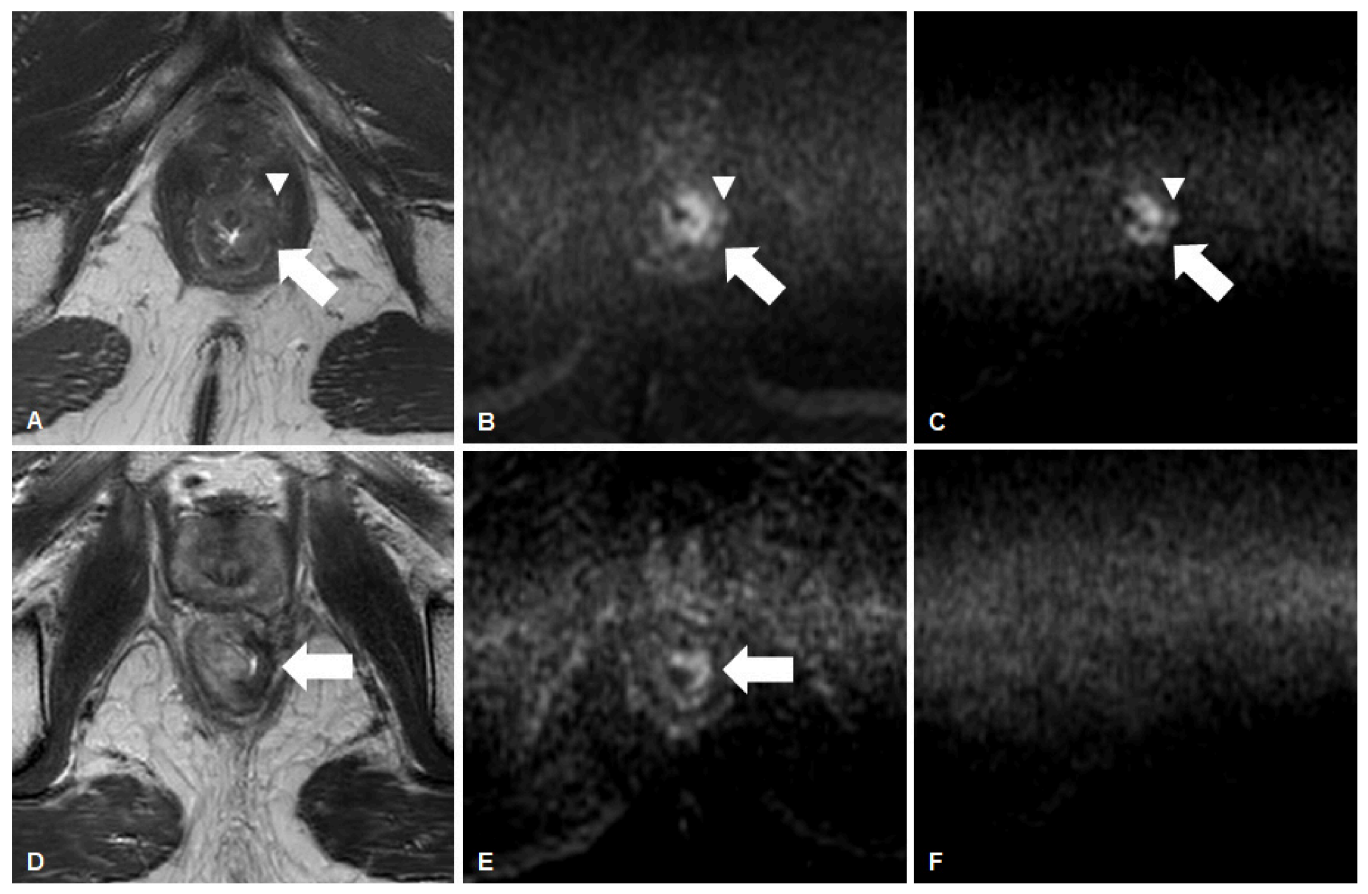
MR images from a 50-year-old male with lower rectal cancer and pathologic stage of ypT2N1, showing the inherent limitation of DWI_b2000_ in detecting near-complete response (near-CR): (**A**) Pre-nCRT T2WI shows eccentric wall thickening involving the lower rectum (arrow) with invasion of internal sphincter (arrowhead). (**B**,**C**) Pre-nCRT DWI_b1000_ and DWI_b2000_, respectively, show diffusion restriction in the corresponding tumor (arrows). Arrowheads mark the same location as in (**A**). (**D**) Post-nCRT T2WI reveals tumor shrinkage with dark signal intensity (SI) (arrow), suggesting predominant fibrotic conversion of the tumor. (**E**) Post-nCRT DWI_b1000_ shows residual high SI at the tumor bed (arrow). (**F**) The residual high SI at the tumor bed is no longer apparent on post-nCRT DWI_b2000_. The pathologic regression grade was 1 (near-CR), which indicates microscopically scattered residual tumor cells. Confidence scores (T2WI/T2WI + DWI_b1000_/T2WI + DWI_b2000_): reader 1—3/2/5; reader 2—5/5/5.

**Figure 5:**
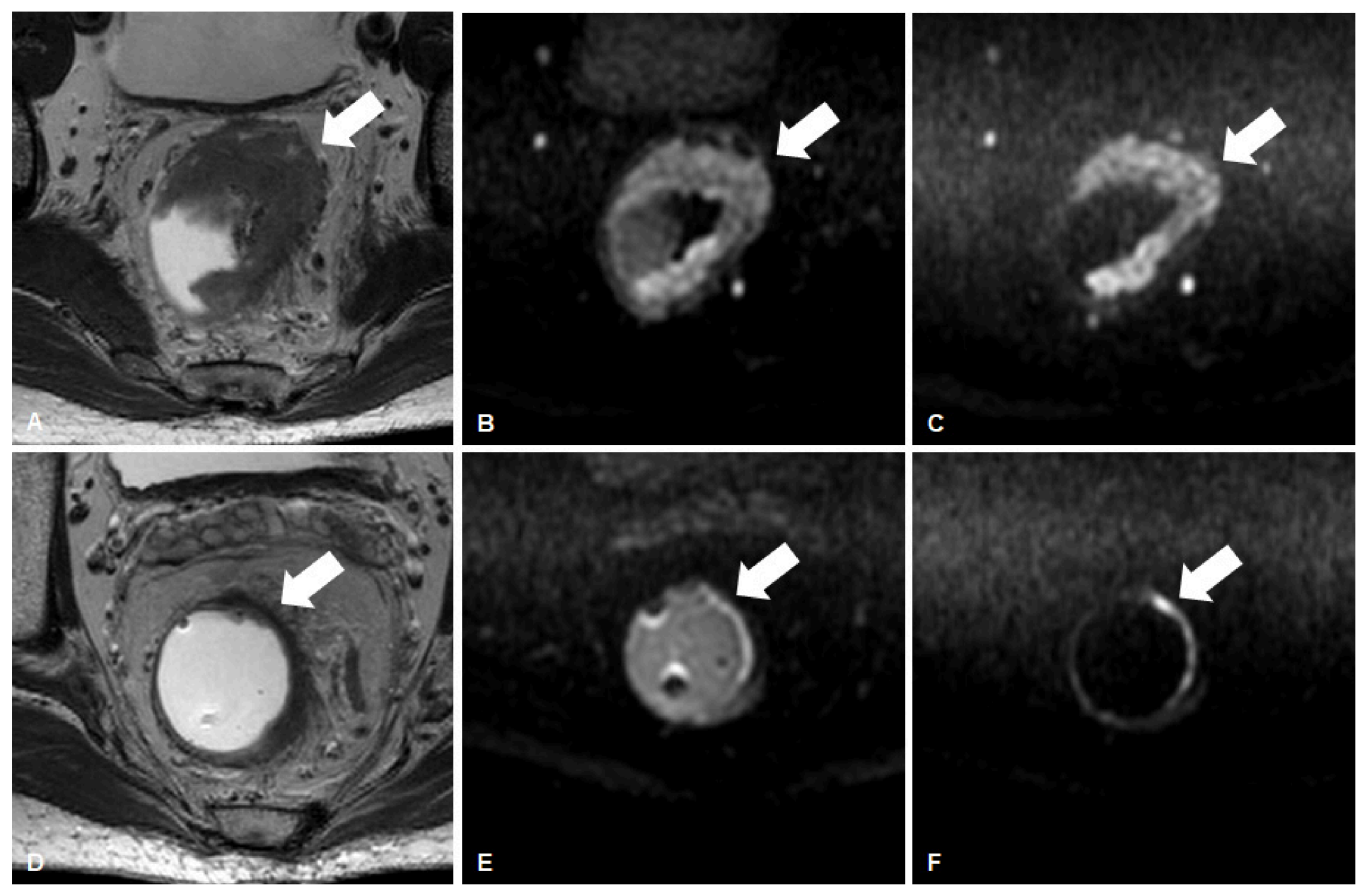
MR images from a 71-year-old male with mid rectal cancer and pathologic complete response, showing a limitation of DWI_b2000_ due to insufficient suppression of post-nCRT inflammatory desmoplastic reactions: (**A**) Pre-nCRT T2WI shows a large semicircular ulcerofungating mass in mid rectum (arrow). (**B**,**C**) Pre-nCRT DWI_b1000_ and DWI_b2000_, respectively, show diffusion restriction in the corresponding tumor (arrows). (**D**) Post-nCRT T2WI shows marked reduction in tumor volume with dark signal intensity (SI) at the tumor bed (arrow). (**E**) Post-nCRT DWI_b1000_ shows a linear high SI along the tumor bed (arrow). (**F**) The linear high SI becomes more conspicuous on post-nCRT DWI_b2000_ (arrow). Histopathologic examination revealed the inflammatory desmoplastic reaction in the completely necrotic tumor bed. Confidence scores (T2WI/T2WI + DWI_b1000_/T2WI + DWI_b2000_): reader 1—3/2/1; reader 2—3/3/1.

DWI_b2000_ also has inherent limitations. False-positive interpretations occasionally occurred in patients with a near-CR, where only scattered microscopic foci of viable tumor cells remained within dense post-treatment fibrosis (Figure 4). In such cases, the cellular density of residual tumor is below the detection threshold of DWI, particularly with the ultra-high *b*-value acquisition, where the reduced signal-to-noise ratio may further limit the visualization of microscopic residual tumor cells. In contrast, we observed that an inflammatory desmoplastic reaction was not sufficiently suppressed even under DWI_b2000_, leading to false-negative interpretations (Figure 5). An inflammatory desmoplastic reaction without tumor cells can retain a degree of SI that mimics residual tumor, especially when the SI from surrounding healthy tissue is completely suppressed [15,34]. Consequently, such inflammatory desmoplastic reactions in patients who have actually achieved a CR could be misdiagnosed as a non-CR with residual tumor.

With regard to false-positive cases associated with a near-CR, the clinical implications of those diagnostic errors may be interpreted in the context of contemporary rectal cancer management. In the current era of total neoadjuvant therapy (TNT), patients achieving a near-CR are increasingly considered for non-operative management [35]. A recent study investigated the outcomes for non-operative management in 61 patients with LARC who achieved a near-CR following TNT and reported a durable clinical response with a 2-year organ preservation rate of 59% and a 2-year overall survival rate of 93.3% [36]. In this setting, microscopic residual tumor cells identified only by pathological examination may not necessarily indicate immediate radical surgery. Although these cases can be misclassified as CRs, the clinical impact may be less critical in light of the recent management shift toward surveillance for this group.

From the perspective of personalized medicine, maintaining high sensitivity for predicting CRs is particularly important when selecting patients for organ-preserving strategies following nCRT. In our study, DWI_b2000_ yielded a sensitivity of 95% for predicting a CR in both readers while demonstrating diagnostic performance with no statistically significant difference compared to that of DWI_b1000_. Notably, the less experienced reader showed a trend toward higher sensitivity with DWI_b2000_ compared with DWI_b1000_ (95% and 79%, respectively). Whether these potential advantages translate into more reliable patient selection or improved oncologic outcomes for non-operative management of LARC remains to be determined in larger prospective studies.

Several limitations should be acknowledged. First, this was a retrospective single-center study; thus, selection bias may not have been completely eliminated. Second, although the decision for non-operative management was made by a multidisciplinary team using digital rectal examination, endoscopy, and restaging MRI, verification bias could not be completely eliminated because MRI, including DWI, contributed to this decision. Third, follow-up of the non-operatively managed patients was relatively short (median: 20 months), and late regrowth may have been missed. Fourth, part of the apparent benefit of DWI_b2000_ may be attributable to suppression of intraluminal gel signal. This benefit may not generalize to centers not using gel. Fifth, the relatively longer acquisition time required for DWI_b2000_ may limit its practical implementation; this limitation may potentially be solved by the use of computed DWI (cDWI), where high *b*-value images are mathematically extrapolated from at least three lower *b*-values. In a previous study, acquired and cDWI_b2000_ showed comparable performance in tumor detection, suggesting that cDWI_b2000_ could potentially replace acquired DWI_b2000_ to save time while maintaining diagnostic performance [37]. Finally, while we compared DWI_b1000_ and DWI_b2000_, the optimal *b*-value for rectal cancer restaging remains to be established through further studies.

## 5. Conclusions

In conclusion, incorporation of DWI_b2000_ into HR-T2WI significantly improves the diagnostic performance for predicting a CR compared with HR-T2WI alone in patients with LARC following nCRT. No statistically significant difference was detected between DWI_b1000_ and DWI_b2000_. Our observation may aid the physician’s proper selection of surveillance candidates for “watch-and-wait” management based on a personally customized approach in the era of TNT.

## Figures and Tables

**Table 1:** MR sequence parameters.

Parameters	T2-Weighted Axial, Sagittal, and Coronal Imaging	DWI (*b* = 0, 100, 1000, 2000 s/mm^2^)
TR (ms)	2500–3783	12,000
TE (ms)	90	75
ETL	15, 15, 14	73
Slice thickness (mm)	3, 5, 4	2
Slice gap (mm)	0.3, 1, 0.4	0
Matrix size	316 × 280, 356 × 331, 332 × 316, respectively	120 × 117
NEX	1	1 (*b*0), 1 (*b*100), 8 (*b*1000), 12 (*b*2000)
FOV (mm × mm)	220 × 220, 250 × 250, 250 × 250, respectively	240 × 240
Acquisition time	2 min 18–24 s	13 min 12 s in total
Number of slices	24–40	66

Diffusion-weighted imaging (DWI) was performed using the single-shot echo planar imaging technique. TR, repetition time; TE, echo time; ETL, echo train length; NEX, number of excitations; FOV, field of view.

**Table 2:** Demographic data of the study population.

Characteristics	Study Population (*n* = 55)
Tumor location	
Upper rectum	14 (25%)
Mid rectum	16 (29%)
Lower rectum	25 (45%)
Treatment response ^1^	
Complete response	2 (4%)
Near-complete response	9 (16%)
Partial response	19 (35%)
Poor response	8 (15%)
Clinical complete response ^2^	17 (31%)
Pathologic tumor stage	
ypT0	2
ypT1	1
ypT2	13
ypT3	20
ypT4	2
Pathologic lymph node stage	
ypN0	28
ypN1	7
ypN2	3
Baseline tumor stage ^3^	
cT4	18 (33%)
cT3	30 (55%)
cT2	7 (13%)
Baseline lymph node stage ^3^	
cN0	9 (16%)
cN+	46 (84%)
Baseline MRF involvement ^3^	
Positive	24 (44%)
Negative	31 (56%)
Baseline EMVI ^3^	
Positive	18 (33%)
Negative	37 (67%)

^1^ Tumor regression grading (TRG) system of the American Joint Committee on Cancer (AJCC), 8th edition. ^2^ Clinical complete response was confirmed with endoscopic surveillance for a minimum of 12 months following restaging MRI. ^3^ Baseline tumor and lymph node stage, MRF involvement, and EMVI status are based on pre-treatment MRI scans. MRF, mesorectal fascia; EMVI, extramural venous invasion. Percentages are proportions of the study population (*n* = 55).

**Table 3:** Diagnostic performance among different MR sequences.

	Sequence	AUC (95% CI)	ΔAUC (95% CI)	Diagnostic Accuracy, %	Sensitivity, % (95% CI)	Specificity, % (95% CI)	PPV, % (95% CI)	NPV, % (95% CI)
Reader 1	T2WI	0.806 (0.677~0.900)	+0.149 * (0.057~0.241)	66	100 (82~100)	47 (30~65)	50 (33~67)	100 (81~100)
		*p* = 0.0015 *		*p* = 0.0007 *	*p* = 1 *	*p* = 0.0001 *		
	T2WI + DWI_b1000_	0.955 (0.862~0.992)	−0.037 ^†^ (−0.079~0.006)	93	95 (74~100)	92 (78~98)	86 (64~97)	97 (85~100)
		*p* = 0.0931 ^†^		*p* = 1 ^†^	*p* = 1 ^†^	*p* = 1 ^†^		
	T2WI + DWI_b2000_	0.918 (0.812~0.975)	+0.113 ^‡^ (0.012~0.214)	91	95 (74~100)	89 (74~97)	82 (60~95)	97 (84~100)
		*p* = 0.0288 ^‡^		*p* = 0.0013 ^‡^	*p* = 1 ^‡^	*p* = 0.0003 ^‡^		
Reader 2	T2WI	0.768 (0.654~0.881)	+0.095 * (0.018~0.173)	71	90 (67~99)	61 (44~77)	55 (36~73)	92 (73~99)
		*p* = 0.0162 *		*p* = 0.1094 *	*p* = 0.5000 *	*p* = 0.0078 *		
	T2WI + DWI_b1000_	0.863 (0.764~0.961)	+0.005 ^†^ (−0.070~0.080)	82	79 (54~94)	83 (67~94)	71 (48~89)	88 (73~97)
		*p* = 0.8936 ^†^		*p* = 1 ^†^	*p* = 0.3750 ^†^	*p* = 0.5000 ^†^		
	T2WI + DWI_b2000_	0.868 (0.770~0.966)	+0.100 ^‡^ (0.004~0.196)	84	95 (74~100)	78 (61~90)	69 (48~86)	97 (82~100)
		*p* = 0.0414 ^‡^		*p* = 0.0391 ^‡^	*p* = 1 ^‡^	*p* = 0.0313 ^‡^		

* Comparison between T2WI and T2WI + DWI_b1000_; ^†^ comparison between T2WI + DWI_b1000_ and T2WI + DWI_b2000_; ^‡^ comparison between T2WI and T2WI + DWI_b2000_. AUC, area under the receiver operating characteristic curve; CI, confidence interval; PPV, positive predictive value; NPV, negative predictive value.

**Table 4:** Subgroup analysis of the diagnostic performance confined to the surgical cohort (*n* = 38).

	Sequence	TP	FN	TN	FP	Sensitivity, n/N	Specificity, %
Reader 1	T2WI	2	0	17	19	2/2	47
	T2WI + DWI_b1000_	1	1	33	3	1/2	92
	T2WI + DWI_b2000_	1	1	32	4	1/2	89
Reader 2	T2WI	2	0	22	14	2/2	61
	T2WI + DWI_b1000_	1	1	30	6	1/2	83
	T2WI + DWI_b2000_	1	1	28	8	1/2	78

TP, true positive; FN, false negative; TN, true negative; FP, false positive.

## Data Availability

The data presented in this study are available upon request from the corresponding author. The data are not publicly available due to patients’ privacy.
